# Large-Scale Analysis of Auditory Segregation Behavior Crowdsourced via a Smartphone App

**DOI:** 10.1371/journal.pone.0153916

**Published:** 2016-04-20

**Authors:** Sundeep Teki, Sukhbinder Kumar, Timothy D. Griffiths

**Affiliations:** 1 Wellcome Trust Centre for Neuroimaging, University College London, London, United Kingdom; 2 Institute of Neuroscience, Newcastle University, Newcastle upon Tyne, United Kingdom; Duke University, UNITED STATES

## Abstract

The human auditory system is adept at detecting sound sources of interest from a complex mixture of several other simultaneous sounds. The ability to selectively attend to the speech of one speaker whilst ignoring other speakers and background noise is of vital biological significance—the capacity to make sense of complex ‘auditory scenes’ is significantly impaired in aging populations as well as those with hearing loss. We investigated this problem by designing a synthetic signal, termed the ‘stochastic figure-ground’ stimulus that captures essential aspects of complex sounds in the natural environment. Previously, we showed that under controlled laboratory conditions, young listeners sampled from the university subject pool (n = 10) performed very well in detecting targets embedded in the stochastic figure-ground signal. Here, we presented a modified version of this cocktail party paradigm as a ‘game’ featured in a smartphone app (The Great Brain Experiment) and obtained data from a large population with diverse demographical patterns (n = 5148). Despite differences in paradigms and experimental settings, the observed target-detection performance by users of the app was robust and consistent with our previous results from the psychophysical study. Our results highlight the potential use of smartphone apps in capturing robust large-scale auditory behavioral data from normal healthy volunteers, which can also be extended to study auditory deficits in clinical populations with hearing impairments and central auditory disorders.

## Introduction

Every day, we are presented with a variety of sounds in our environment. For instance, on a quiet walk in the park we can hear the sound of birds chirping, children playing, people talking on their mobile phones, vendors selling ice cream amongst other sounds in the background. The ability to selectively listen to a particular sound source of interest amongst several other simultaneous sounds is an important function of hearing systems. This problem is referred to as the ‘cocktail party problem’ [1, 2, 3, 4]. Auditory cortical processing in real-world environments is a fertile field of scientific pursuit [5], and an inability to perform figure–ground analysis, especially speech-in-noise detection, is one of the most disabling aspect of both peripheral hearing loss and central disorders of hearing [6, 7].

Previous laboratory-based research on auditory scene analysis employed synthetic stimuli that are conventionally based on simple signals such as pure tones, sequences of tones of different frequencies, or speech-in-noise for instance [8, 9, 10]. We designed a stimulus that consists of a series of chords containing random frequencies that change from one chord to another. The stimulus, referred to as the Stochastic Figure-Ground (SFG) signal, has some common features with previous informational masking (IM) stimuli in which masking is produced by multiple elements that do not produce energetic masking at the level of the cochlea [11, 12, 13]. Unlike previous IM stimuli there is no spectral ‘protection region’ around the target: in the SFG paradigm subjects are required to separate complex figures with multiple frequencies from a noisy background over the same frequency range. The SFG stimulus comprises of a sequence of chords that span a fixed frequency range, and the pure tones comprising the chords change randomly from one chord to another. We incorporated a target in the middle of the signal that contains a specific number of frequencies (where the number of frequencies is referred to as the ‘coherence’ of the stimulus) that repeat for a certain number of chords (referred to as the ‘duration’ of the stimulus). The SFG stimulus offers better parametric control of the salience of the figure (e.g. by changing the coherence, duration, size and density of chords) as demonstrated in previous psychophysical experiments [14, 15]. The stimulus requires the grouping of multiple elements over frequency and time, similar to the segregation of speech from noise. However, unlike speech-in-noise paradigms, segregation in the SFG stimulus depends on the temporal coherence of the repeating components [15]

This paradigm, however, has only been tested in traditional laboratory settings based on limited numbers of participants (usually 10–15) who are typically undergraduate students from local universities. While this represents the conventional approach for psychophysical experiments, the recent emergence of web-based and app-based experimentation has the potential to provide large amounts of data from participants with diverse demographic and hearing profiles. In order to examine auditory segregation performance from a large and diverse pool of subjects, we customized our figure-ground segregation task [14, 15] as a short engaging game for ‘The Great Brain Experiment’ (www.thegreatbrainexperiment.com), a large-scale cognitive science crowdsourcing app [16] developed for iOS and Android based smartphones and tablets in association with the Wellcome Trust, UK.

On every trial, participants were required to indicate via a button press, which one of the two SFG stimuli contained a target. We fixed the coherence of the figure (to 8 repeating frequencies) and varied the duration of the figure (12, 8, 6, 4 and 2 chord segments) in five increasingly difficult levels. The main aim of the experiment was to examine the utility of the app in terms of studying auditory segregation behavior.

Another aim of the study was to assess segregation behavior as a function of age. Aging is accompanied by changes in the peripheral auditory structures, resulting in poorer detection thresholds typically at high frequencies [17]. It represents a major challenge for hearing science and audiology because of the significant impact on the quality of life and lack of targeted treatments. Aging results in a loss of hearing acuity due to the inability to use a combination of auditory cues, including frequency and duration [18], spatial cues [19], temporal regularity [20], and the inability to process sequential order of stimuli [21], melodies [22], and understand speech in noisy background [23]. However, not all auditory deficits have a cochlear basis, and may have a central origin, that may result in impaired understanding of the acoustic world due to the higher-level deficits related to attention and working memory. Here, we predicted that older participants (50 years and above) would be impaired at the task compared to younger participants (18–29 years) due to poor spectrotemporal integration that is necessary to extract the temporally coherent targets. However, given the lack of systematic controls whilst playing the app, the precise nature of such a deficit cannot be accurately determined.

The use of the app allows large-scale powerful studies to examine the effect of demographic variables such as age [24] and hearing loss on figure-ground analysis. The present study represents a proof of concept in which we demonstrate that behavior measured via the smartphone app is consistent with our previous psychophysics results [15].

## Materials and Methods

### Smartphone app

Our auditory figure-ground segregation experiment (‘How well can I hear?’), is one of a suite of eight psychological paradigms featured in the app, The Great Brain Experiment launched by the Wellcome Trust Centre for Neuroimaging, University College London in collaboration with an external developer (White Bat Games). Initially launched as a public engagement and crowdsourcing app for iOS and Android devices in March 2013, the original release comprised of four games based on working memory, attention, response inhibition and decision-making [16]. Funded by the Wellcome Trust, the app received widespread media attention and quickly garnered several thousands of participants and user plays. Building upon the success of the initial release, we designed our auditory game for the next release launched on November 21, 2013.

**Fig 1:**
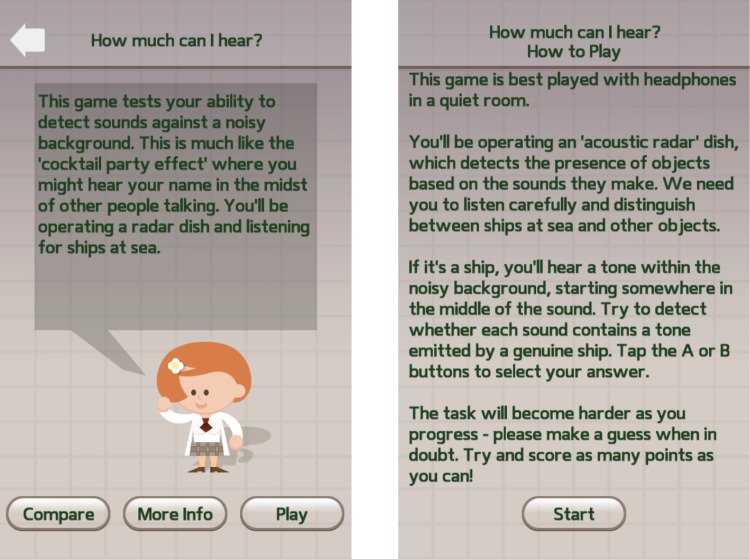
Smartphone game task instructions. Participants are shown two screenshots that explain the scientific rationale of the game (left) and the context of the game and specific task instructions (right).

The study was approved by the University College London Research Ethics Committee (application number: 4354/001). Once downloaded, the participant was instructed to fill a brief demographic questionnaire (age, gender, educational status, location, native language, current life satisfaction level) and provided written informed consent. At the start of each game, the participant received brief information about the scientific principles underlying the game (Fig 1A) as well as detailed information about how to play the game (Fig 1B). Participants could play a game any number of times and the number of plays was recorded, however, only the first complete play was registered as a response. Once the game was completed, a dataset was submitted to the server (provided stable internet connection) with game-specific information and responses. The device used to submit the first play of each participant was assigned a unique ID number (UID) and subsequent plays from that device were tagged with the same UID. However, no personal identification was recorded. At the end of each play, the participant received his/her percentile score.

### Stimuli

The SFG stimulus comprises of a sequence of chords containing a random number of pure-tone components that are not harmonically related. A subset of these components is repeated identically over a certain number of chords, resulting in the spontaneous percept of a coherent ‘figure’ emerging from the random background. The appearance of the figure embedded in randomly varying background simulated the perception of an auditory object in noisy listening environments. Crucially, the figure can only be extracted by integrating along both frequency and time dimensions. We refer to the number of repeating components that comprise the figure as the ‘coherence’, and the number of chords over which they repeat as the ‘duration’ of the figure [14, 15].

**Fig 2:**
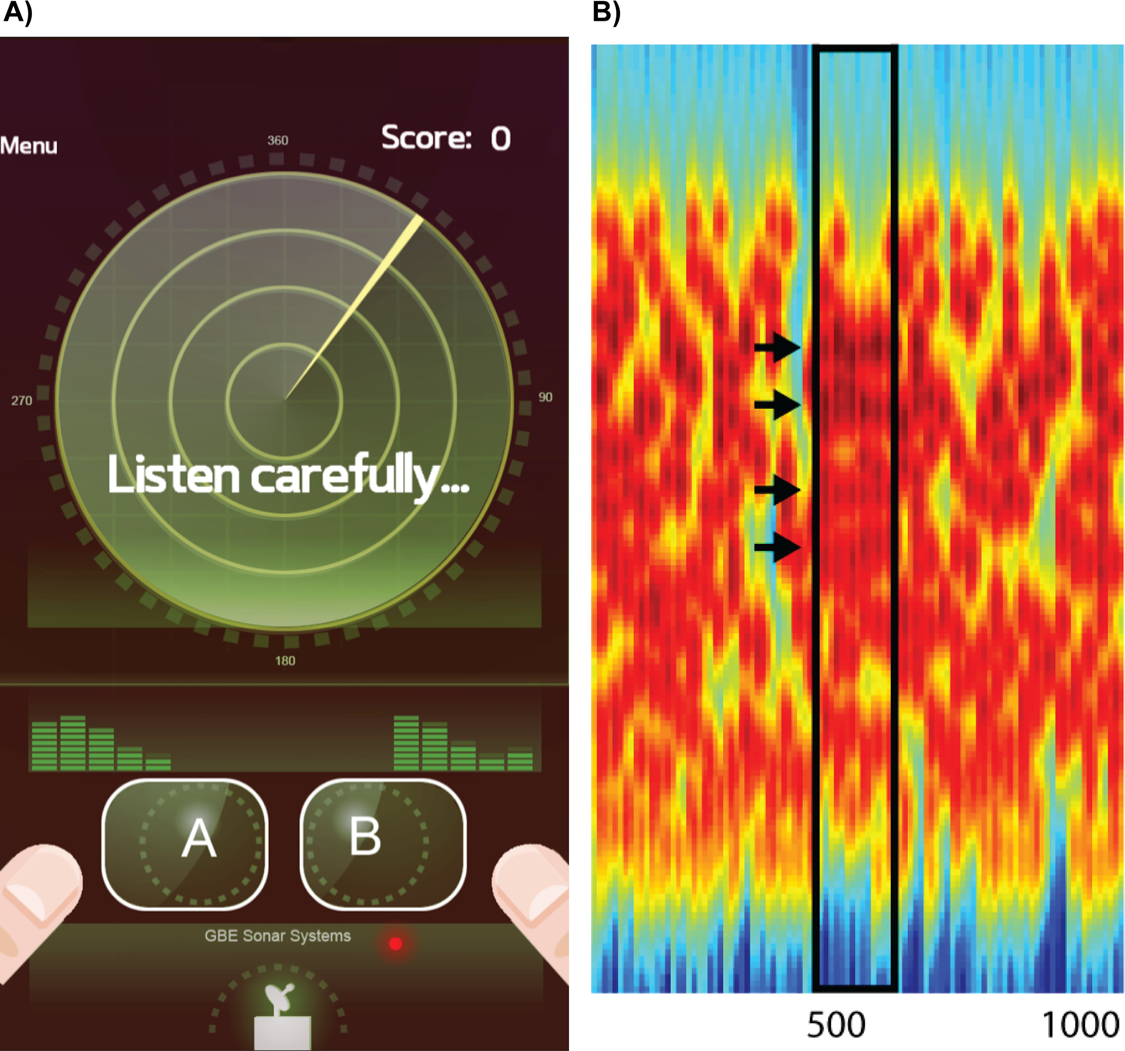
Task and stimulus. (A) During the game, the participants are shown a radar screen (left) and are required to listen to two sounds (marked A and B). The participants’ task is to judge which of the two sounds contained a target. (B) The spectrogram of a SFG sound containing a target with repeating components depicted by the black arrows is shown on the right. Each sound is 1s long and spans a frequency range from 0.2–2.1kHz.

In the present study, the SFG signal comprised of forty 25ms chords equal to 1s duration (Fig 2B; coherence equal to 4 here for illustration purposes only, actual value used in the experiment = 8). The range of frequencies in the stimulus was reduced to 0.2–2.1kHz (from 0.2–7.2kHz; [14, 15]) due to the restrictions imposed by the sound card in the smartphone devices. The coherence of the figure, i.e., the number of repeating components was fixed at 8 and the onset of the figure was also fixed at 0.4s post-stimulus onset. The duration of the figure, i.e. the number of chords over which the coherent components repeat, was selected from one of five values: 12, 8, 6, 4, and 2, corresponding to the different levels of the game. The stimuli were created at a sampling rate of 44.1kHz in MATLAB 2013b (The MathWorks Inc.) and drawn from a set of 16 different wav files for each figure duration and target condition (figure present or absent).

### Procedure

The experiment was presented as a radar detection game where the participant assumed the role of a radar operator. Before the game started, the instruction screen prompted the participant to play the game with headphones. The task required the participant to decide whether the acoustic mixture contained a signal corresponding to the target sound of a ship or not by pressing one of two buttons on the device’s touchscreen (Fig 2A). Every trial consisted of two 1-s long SFG stimuli, where one of them contained the figure while the other did not. The order of the stimuli was counterbalanced on each trial. Feedback was provided after each trial. The game consisted of five levels with five trials each corresponding to different duration values: 12, 8, 6, 4, and 2. The game started at an easy level—with the number of repeating components equal to 12. Each level consisted of 5 trials and if the participant scored more than 40% (i.e., > 2/5 correct responses) on that level, the game proceeded to the next more difficult level with a lower duration value. On average, the game took approximately 5 minutes to complete. The score was scaled according to the difficulty level: a correct response at levels 1–5 (duration of figure: 12, 8, 6, 4, 2) was equal to 1, 2, 3, 4, and, 5 points respectively. At the end of the game, the participant received the final score (maximum score: 75).

In contrast, the experimental paradigm in the psychophysical study [15] was slightly different: the SFG stimulus with 25ms chords that had a broader frequency range (0.2–7.2kHz), the coherence (values: 1, 2, 4, 6, 8) and the duration (values: 2–10) of the figure were varied in a block design (50 trials per block), and the onset of the figure was jittered. The hit rates for the condition corresponding to the values tested in the game (coherence of 8 and duration of 2, 4, 6, and 8) are reported for comparison.

### Data analysis

Data from all participants and all plays were collated as a comma separated value file on the app server. The relevant fields in the data were imported into MATLAB R2014b (MathWorks Inc.) for analysis using custom scripts. The primary fields of interest included the score (hit = 1, miss = 0) and response time for each of the 25 trials. Demographic information including age, gender, educational status, and type of device was also extracted. The dataset presented in this study features plays collected over the period of a year, from the launch of the game on November 21, 2013 to December 31, 2014. In all, 14451 participants (6309 females; 8142 males) played the game 33715 times (mean number of games player per participant: 2.33, standard deviation: 3.96; range: 1–68). 51.47%, 25.80%, 10.66%, 4.80% and 2.36% of all participants played the game only once, only twice, only thrice, only four and only five times respectively. The percentage of participants who played the game more than 5 times was only 4.91%.

**Fig 3:**
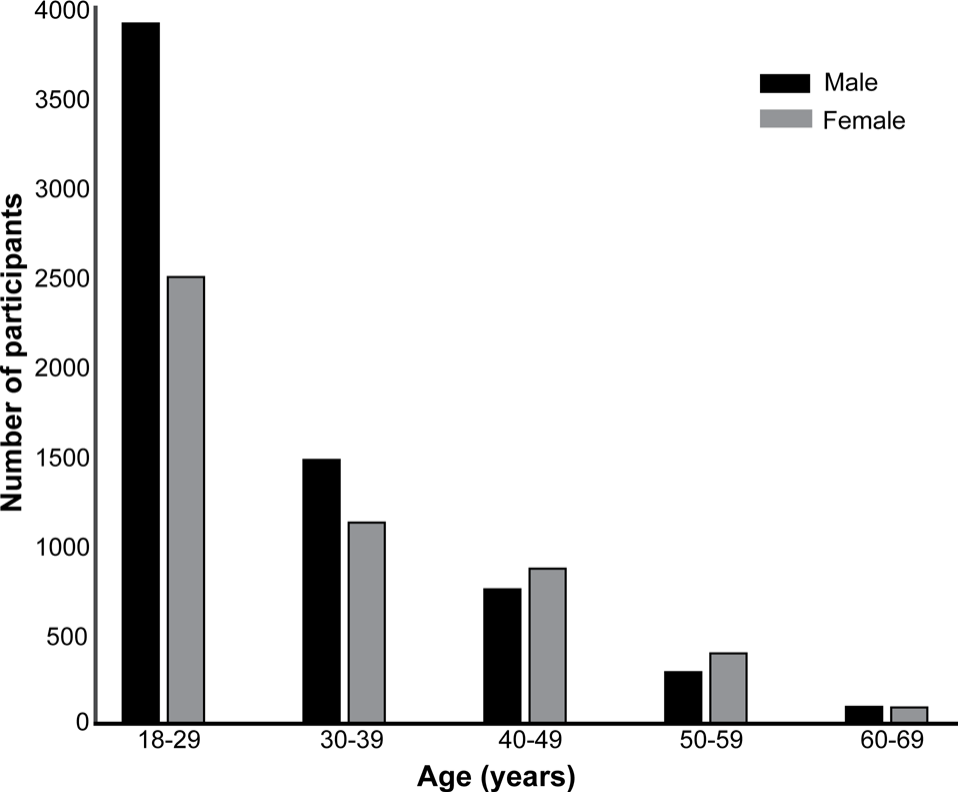
Demographics. Bar charts show the number of participants according to different age groups and gender (male in black bars, female in grey bars).

The data were subjected to a few rigorous exclusion criteria—data from participants below 18 years of age were rejected as well as data where the game stopped when the participant scored less than or equal to 40% on two consecutive levels (i.e. correct score of 2 or less on the 5 trials in each level). The latter criterion applied to all games featured in the app. After removing the data from participants younger than 18 years, 11489 valid participants were obtained which further reduced to 7049 after rejecting participants on the basis of poor performance as described above. The response times were also measured for each trial and any game with a response time greater than 6s for a single trial was also excluded, finally resulting in 5148 (2093 females; 3055 males) valid participants. We used a conservative threshold for reaction times (compared to psychophysical experiments) to account for variations in terms of touch screen response fidelity, device type and the uncontrolled context in which the participants played the game. The majority of the resultant participant group (77.2%) spoke English as their native language. 2849 (2299) participants used iOS (Android) devices to play the game. In terms of educational qualifications, the number of participants educated at the level of GCSEs, A-levels, Bachelor’s, and Postgraduate education was equal to 579, 1096, 2230, and 1243 respectively. The age and gender distributions for the valid participants are shown in Fig 3.

### Statistical analysis

The main analysis focused on examining the effect of the duration of the figure on the performance (hit rate) and the response time as well as age-related differences in performance and response times. All statistical tests were conducted in MATLAB R2014b using in-built functions in the statistics toolbox. Sphericity was evaluated using the Greenhouse-Geisser correction. Effect sizes (partial eta squares: η2) were analyzed using the Measures of Effect Size toolbox in MATLAB [25].

## Results

The scores and reaction times of the valid participants were tested for normality in order to justify the use of parametric tests. The scores were normally distributed (Shapiro-Wilk W = 0.997, p < 0.001) with a mean of 43.47 (maximum score being 75) and standard deviation of 8.01. The response times had a mean value of 1.12s and a standard deviation of 0.79s and were log-transformed to ensure normality (Shapiro-Wilk W = 0.98, p < 0.001).

**Fig 4:**
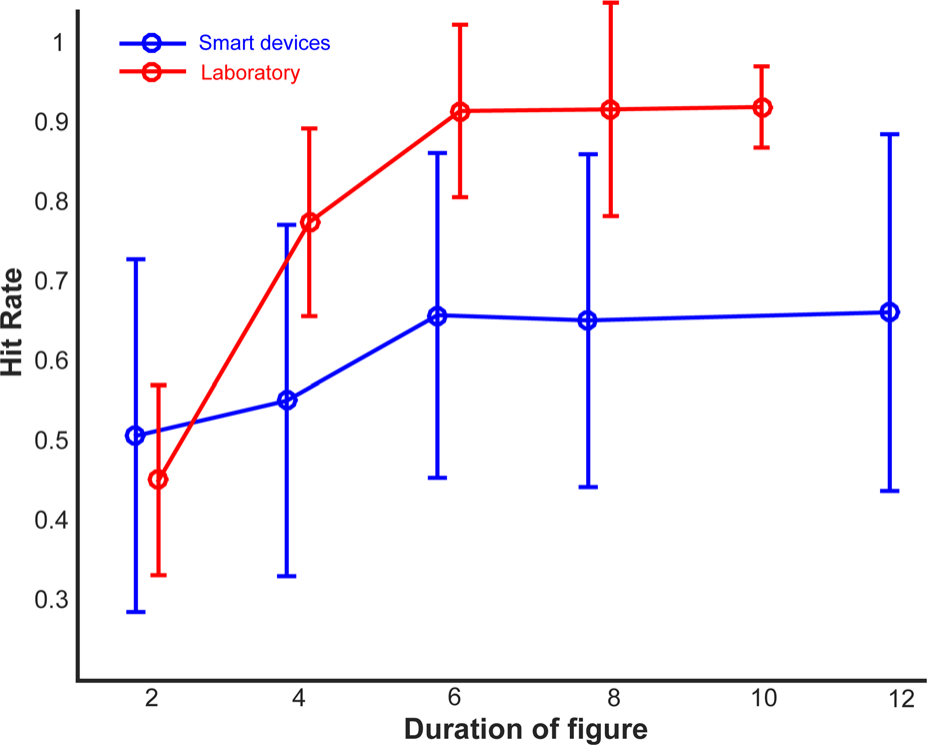
Performance in the app vs. the psychophysics study. Hit rates are plotted as a function of the duration of the figure for data obtained from the smartphone app (n = 5148, in blue) and the psychophysics study (n = 10, in red). Error bars depict 1 STD.

The aim of the analysis was to determine the effect of the duration of the figure on hit rates and reaction times. We observed a main effect of duration on the hit rate: F(4,25735) = 571.9, p <0.001, η2 = 0.082. The hit rate for duration of 2 was at chance (0.51) and increased monotonically for duration values of 4 (0.55) and 6 (0.66) and then remained almost constant for durations of 8 and 12 (0.65 and 0.66 respectively) as shown in Fig 3 (blue). The pattern of responses (Fig 4, blue) is remarkably similar to the hit rates observed in the psychophysical experiment (Fig 4, red) where hit rates increased monotonically from duration of 2 (0.45) to 4 (0.78) and then leveled off for higher duration values (0.92). The drop in performance based on the game was ~25% on average which can be attributed to the greater amount of noise in the data, given the uncontrolled acoustic and experimental settings. However, the effect size obtained in the smartphone study was approximately equal to the effect size observed for similar range of coherence (equal to 8) and duration values (2–10) in the psychophysical experiment (η2 = 0.081).

**Fig 5:**
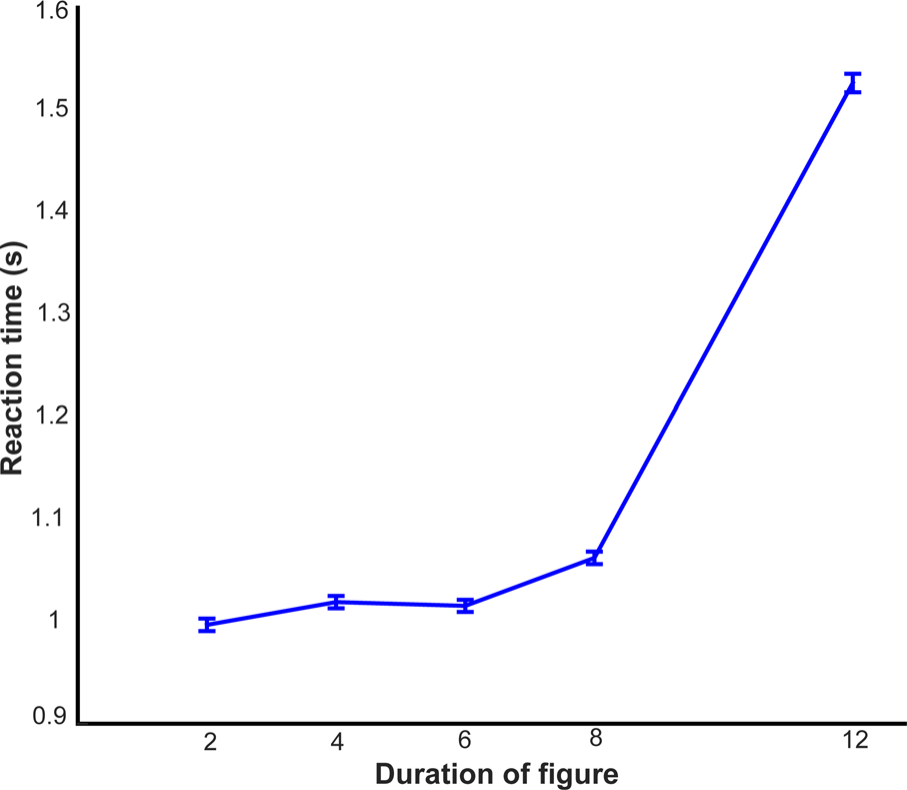
Reaction Times. Reaction times (in seconds) are plotted against the duration of the figure. Error bars depict 1 SEM.

We also observed a significant effect of duration on the response times: F(4,25735) = 933.23, p <0.001, η2 = 0.127 (Fig 5). The response times were highest for the first trial: 2.58 +/- 1.61s, presumably because the first trial could be perceived to be the most difficult given the lack of adequate practice. With increasing number of trials, the response times stabilized and for trial numbers 6–25, the response times ranged from 0.99s to 1.10s.

**Fig 6:**
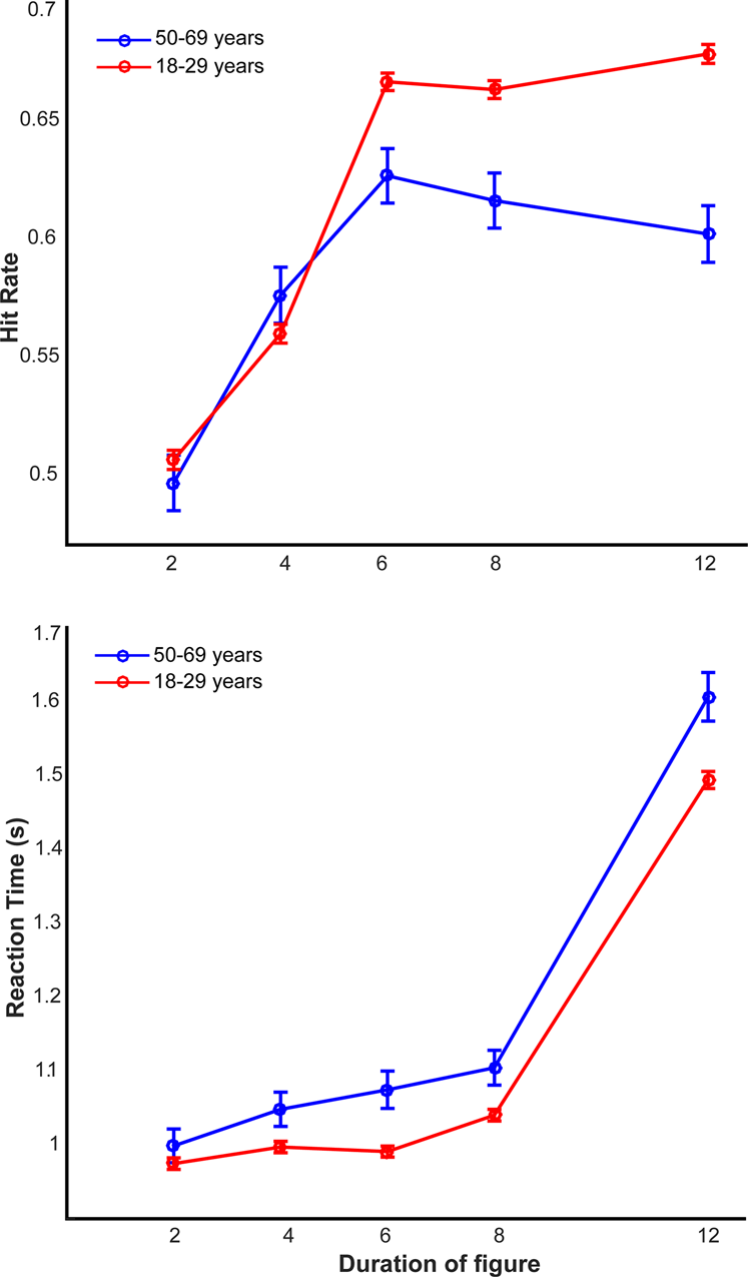
Performance and reaction times for two different age groups. (A) Hit rates are plotted for two different age groups: 18–29 year olds (n = 3033, in blue) and 50–59 year olds (n = 324, in red). (B) Reaction times for the younger and older set of participants as above are shown in blue and red respectively. Error bars depict 1 SEM.

Finally, we also analyzed performance and response times as a function of age and focused on two age groups: 18–29 year olds (n = 3033) and 50–69 year olds (n = 324). The hit rates of the two groups are plotted in Fig 6A. We performed an ANOVA with group as a between-subject factor (young vs. old participants) and duration of the figure as a within-subject factor. We found a significant main effect of group: F(1,3374) = 20.10, p < 0.001 but no significant effect of duration: F(4,13496) = 0.74, p = 0.56, nor any significant interaction: F(4,13496) = 1.51, p = 0.20. Although the interaction between age and duration was not significant (p = 0.20), we performed an exploratory analysis by performing post hoc t-tests for each level of duration: we observed a significant difference in the hit rate only for durations of 12 (t = 5.92, p < 0.001, df = 3374), 8 (t = 3.84, p = 0.0001, df = 3374), and 6 (t = 3.34, p = 0.0008, df = 3374) and not for the more difficult duration conditions of 4 (t = -1.28, p = 0.2, df = 3374) and 2 (t = 0.75, p = 0.46, df = 3374).

Fig 6B shows the response times for the two age groups. A similar ANOVA analysis yielded a significant main effect of group: F(1,3374) = 20.01, p < 0.001, a main effect of duration: F(4,13496) = 4.40, p = 0.001 as well as a significant interaction: F(4,13496) = 3.78, p = 0.004. Post-hoc t-tests confirmed a significant difference in response times for all but the smallest duration value: 12 (t = 3.05, p = 0.002, df = 3374), 8 (t = 2.52, p = 0.01, df = 3374), and 6 (t = 3.41, p = 0.0006, df = 3374), 4 (t = 2.05, p = 0.04, df = 3374), 2 (t = 0.96, p = 0.34, df = 3374).

## Discussion

We demonstrate that experiments to assess a high-level aspect of auditory cognition using an established auditory paradigm can be replicated using an app, despite the uncontrolled testing environment compared to the laboratory. We present data from 5148 participants, gathered over the course of a year. Our particular game was co-launched with three other games featured in the Great Brain Experiment app. Presented as a citizen science project (e.g. [26, 27]), the app was successful in attracting tens of thousands of users through online and social media forums because of its scientific appeal and interactive gamification of psychological experiments. We observed that results from our auditory segregation game were consistent with results from laboratory experiments and highlight the potential use of such citizen science projects in engaging with the public and replicating laboratory experiments based on a large and diverse sample of participants.

The task was based on a stochastic figure-ground (SFG) stimulus developed to simulate segregation in complex acoustic scenes [14, 15]. Unlike simple signals used to study segregation, like the two-tone alternating streaming stimulus [2, 3], or informational masking paradigms [11, 12], segregation in the SFG stimulus can only be achieved by integrating across both frequency and time [15, 28]. We have demonstrated that listeners are highly sensitive to the emergence of brief ‘figures’ from the random ongoing background, and that performance is significantly modulated as a function of the coherence and duration of the figure. Additionally, we also showed that our behavioral data [15] are consistent with the predictions of the temporal coherence theory of auditory scene analysis [29, 30]. The temporal coherence model is based on a mechanism that captures the extent to which activity in distinct neuronal populations, that encode different perceptual features, is correlated in time.

We used this paradigm to study auditory segregation as the SFG stimulus is associated with a short build-up (figure duration varied from 50-350ms in original experiments) whereas for streaming signals, build-up takes 2-3s in quiet environments [31, 32, 33]. The build-up of auditory segregation represented an important practical factor related to the overall time required to complete the experiment. With a short build-up for the SFG signal, the task took approximately 5 minutes. However, streaming paradigms would have taken much longer to complete, given the slower build-up that may be further accentuated by the lack of a controlled acoustic environment when playing on the app.

We fixed the number of repeating components, i.e. the coherence of the figure, to eight and varied the number of chords over which they repeat. Performance was found to vary significantly with the duration of the figure, replicating our earlier results [14, 15]. We also found a main effect of duration on the response times, i.e. response times decreased with increasing duration of the figure. Compared to the results obtained from the psychophysical studies, performance on the app was significantly impaired. This can be due to a number of reasons related to the differences in experimental design (1-alternative forced choice design for the laboratory experiments vs. 2-alternative forced choice design for the app), experimental setup (laboratory experiments were conducted in soundproof booths with participants listening to the stimuli over headphones at controlled sound levels, whilst there was no such experimental or acoustic control for the app), as well as differences in training and practice of the experimental task (participants in the psychophysics studies received adequate instruction about the stimuli and also got to practice the task whilst the app only had minimal instruction about the scientific rationale of the study and response instructions).

Data from the app is associated with greater within-subject measurement noise that is reflected in lower hit rates as well as greater between-subject noise that is reflected in the higher variance in the population data (see Fig 4). Thus, compared to standard psychophysical settings, experimenting on the app is associated with greater noise both at the input level (sensory signal) as well as at the output level (response).

The large sample of participants allowed us to analyze segregation behavior as a function of age. Based on previous research (see introduction), we expected older participants (aged 50–69 years) to be worse on the task than a younger cohort (18–29 years). Performance accuracy as well as response times were modulated by age, i.e. we found significantly lower hit rates and longer response times in the older versus the younger participants. Although peripheral hearing suffers with normal aging, e.g. due to loss of hair cells and spiral ganglion neurons [34], there are multiple factors that contribute to poor scene analysis abilities in older adults. Aging affects frequency resolution, duration discrimination, spatial localization, melody perception as well as speech comprehension in noisy backgrounds [18, 19, 20, 21, 22, 23]. In our experimental paradigm, we have previously demonstrated that segregation of the figures from the background relies on temporal coherence [15]. Recent work has demonstrated that in addition to spectral features, temporal coherence is a vital cue in scene analysis and promotes integration whilst temporal incoherence leads to segregation [35, 36, 37]. Our results provide the first demonstration that the use of temporal coherence as a cue for scene analysis may worsen with age, a result that needs to be confirmed in proper psychophysical settings. However, since the neural substrate of temporal coherence analysis is not yet known [29], it is difficult to ascertain whether the behavioural deficit with aging is associated with peripheral or central auditory pathways.

Although smartphone experiments are useful in gathering data from a large number of potentially very diverse participants, and link datasets across time and tasks with user specific IDs, their use must be considered carefully according to the needs of the study. Recruitment of participants via an app can be a potentially demanding task, requiring constant use of press and social media to attract new users. For a smaller number of participants (e.g. a few hundred), web-based testing or recruitment through Amazon’s Mechanical Turk [38] may be more beneficial. The cost of developing an app represents the main cost, as it is best outsourced to professional developers. Another drawback is the limited technical specifications that can be harnessed on a smartphone, as opposed to web-based experiments or laboratory experiments run on computers or laptops. Nevertheless, the benefits outweigh the limitations and previous work based on this app [16, 39, 40] highlights the advantages of running large-scale experiments that show consistent results with experiments conducted in the laboratory. Apart from scientific applications, such app based games are also an effective means of engaging the public with scientific research.

In summary, we demonstrate that standard psychoacoustic experimental results can be replicated effectively using smartphone apps. The observed effect sizes are similar given the noisy and limited data points and the uncontrolled acoustic and experimental environment. These results highlight the utility of smartphone applications for collecting large-scale auditory behavioral data for basic research and clinical investigations as well.
